# Supplementary material for: Preparation and Characterization of Cellulose Nanocrystals from Bamboos and Their Application in Cassava Starch-Based Film
Source: Polymers (Basel). 2023 Jun 8;15(12):2622. doi: 10.3390/polym15122622 (PMC10301340; doi:10.3390/polym15122622)
Supplement: Supplementary file 1 [file polymers-15-02622-s001.zip › polymers-2346283-supplementary.pdf]

Supplementary data

Preparation and Characterization of Cellulose Nanocrystals from Bamboos and Its Application in Cassava Starch-Based Film

Parichat Thipchai, Winita Punyodom, Kittisak Jantanasakulwong, Sarinthip Thanakkasaranee, Sasina Hinmo, Kanticha Pratinthong, Gopinath Kasi, Pornchai Rachtanapun*

Table S1. The raw data for chemical constituent calculation.

Chemical constituents (%)	Samples	1	2	3	AVG	SD
Holocellulose (%)	TSG	70.26	70.26	71.26	70.59	0.58
	DSM	73.95	74.95	75.02	74.64	0.60
	BL	72.36	73.96	73.00	73.11	0.81
	BS	63.48	64.58	65.25	64.44	0.89
α-cellulose (%)	TSG	40.51	39.97	40.22	40.23	0.27
	DSM	42.05	43.53	42.01	42.53	0.86
	BL	41.04	41.05	43.01	41.70	1.13
	BS	36.85	38.42	37.05	37.44	0.85
Hemicellulose (%)	TSG	29.75	30.29	31.04	30.36	0.65
	DSM	31.90	31.42	33.01	32.11	0.81
	BL	31.32	32.91	29.99	31.40	1.46
	BS	26.63	26.17	28.19	27.00	1.06
Lignin (%)	TSG	23.21	22.34	22.02	22.52	0.61
	DSM	22.92	23.22	24.02	23.39	0.57
	BL	29.35	28.22	29.00	28.86	0.58
	BS	24.37	25.15	23.79	24.44	0.68
Extractive (%)	TSG	2.99	3.20	3.06	3.08	0.11
	DSM	4.04	4.15	4.78	4.32	0.40
	BL	3.24	3.12	3.09	3.15	0.08
	BS	5.06	4.93	4.82	4.94	0.12
Ash (%)	TSG	3.60	3.02	3.73	3.45	0.38
	DSM	1.89	1.77	2.00	1.89	0.12
	BL	2.06	2.02	1.92	2.00	0.07
	BS	2.11	2.48	2.30	2.30	0.19

Table S2. Peak analysis data for XRD calculation of crystallite sizes of cellulose and CNC from four bamboo species.

Samples	Peak position (2theta)	FWHM	Beta	Cos Theta	Crystallite sizes
Cellulose-TSG	22.02	2.95	0.05	0.98	2.86
Cellulose-DSM	22.14	2.98	0.05	0.98	2.84
Cellulose-BL	21.68	4.00	0.07	0.98	2.11
Cellulose-BS	21.66	3.61	0.06	0.98	2.34
CNC-TSG	21.88	6.71	0.12	0.98	1.26
CNC-DSM	21.76	5.15	0.09	0.98	1.64
CNC-BL	21.27	5.61	0.10	0.98	1.51
CNC-BS	21.76	6.24	0.11	0.98	1.35
